# An open-source platform for measuring optomotor responses in mice

**DOI:** 10.1016/j.jphyss.2026.100113

**Published:** 2026-10-23

**Authors:** Naofumi Suematsu, Toshihide Ikeda, Takanori Aoki, Moe Asayama, Takuro Horii, Atsushi Hoshino, Hiroshi Tanaka, Shogo Soma

**Affiliations:** a Department of Molecular Cell Physiology, Kyoto Prefectural University of Medicine, Kyoto, 602-8566, Japan; b Department of Ophthalmology, Kyoto Prefectural University of Medicine, Kyoto, 602-8566, Japan; c Laboratory of Genome Science, Biosignal Genome Resource Center, Institute for Molecular and Cellular Regulation, Gunma University, Gunma, 371-8512, Japan; d Department of Cardiovascular Medicine, Kyoto Prefectural University of Medicine, Kyoto, 602-8566, Japan

**Keywords:** Open source, Optomotor response, Visual acuity, Contrast sensitivity, Retinitis pigmentosa, RHO-P23H mouse, Python, PsychoPy

## Abstract

The optomotor response (OMR) is a valuable behavioral index for assessing visual function in basic research; however, commercially available OMR evaluation equipment is expensive and lacks flexibility, as proprietary software and fixed hardware geometry restrict custom stimulus protocols and system modifications. Alternative low-cost, non-OMR methods often require extensive animal pre-training and are unsuitable for high-throughput studies. To overcome these limitations, we developed a low-cost, open-source OMR recording system for mice that lowers both financial and practical barriers, thereby facilitating widespread adoption across laboratories. Following a one-hour training session for evaluators, the system enabled precise quantification of visual acuity and assessment of contrast sensitivity in normal-sighted mice, detection of age-related visual decline, and evaluation of OMR extinction in retinitis pigmentosa model mice. This system facilitates accessible and efficient assessment of visual function in vision research.

## Highlights

• Low-cost, open-source OMR system was developed to assess visual function in mice.
• Brief evaluator training ensured high inter-observer and test–retest stability.
• Precise quantification of visual acuity and contrast sensitivity was achieved.
• We detected age-related visual decline and OMR extinction in disease models.
• Our system facilitates accessible, high-throughput vision research with minimal cost.

## Introduction

Visual impairment caused by retinal and neuro-vascular diseases represents a major global health burden [1], [2]. In contrast to the remarkable progress in therapeutic approaches such as differentiation from ES/iPS cells, organoid technologies, viral vector–based innovations, and nanotechnology in animal models [3], [4], [5], [6], relatively little progress has been made in simplifying and broadening access to methods for evaluating visual performance. Conventional evaluation systems for rodent visual acuity, like water maze and visual-cue detection system [7], [8], [9], [10], [11], [12], require extensive animal pre-training, making it difficult to chronically evaluate the visual performance during the juvenile period or the intervention effects from the beginning of the operation. Meanwhile, although the assessment of the optomotor response (OMR) does not require the animal training because it relies on the animal's natural reflex to visual stimuli [13], [14], [15], [16], [17], commercially available equipment is still expensive and many investigators cannot conduct high-throughput evaluations.

To overcome these limitations, we developed an open-source system for evaluating the mouse OMR. As described below, our system enables precise assessment of visual acuity in normal-sighted mice, as well as standard contrast sensitivity, age-related declines in visual acuity, and OMR extinction in retinitis pigmentosa model mice.

## Materials and methods

All experimental protocols were approved by the Institutional Animal Care and Use Committee of Kyoto Prefectural University of Medicine, and all procedures were carried out according to the Japanese Act on the Welfare and Management of Animals and the Guidelines for the Proper Conduct of Animal Experiments issued by the Science Council of Japan.

Mice were maintained on 12-h light/dark cycles, and all behavioral testing was performed during the dark phase. C57BL/6 mice (Institute for Shimizu Laboratory Supplies, Kyoto, Japan) were used for the following experiments: inter-evaluator reliability testing (males n = 2, females n = 2; Fig. 2A), week-to-week stability test (males n = 3, females n = 2; Fig. 2B,C), contrast sensitivity function assessment (males n = 3, females n = 3; Fig. 2D), and an evaluation of age-related visual decline (9-month-old: males n = 5, females n = 6; 18-month-old: males n = 5, females n = 5; Fig. 3A). Partially humanized Pro23His (P23H) mutant rhodopsin mice (B6;Cg-Rho<em1(P23H)Ahosh> , RBRC12413; RIKEN BioResource Research Center, Tsukuba, Japan) were used as retinitis pigmentosa model mice for confirming their visual impairments (*Rho*^*P23H/P23H*^: 4-month-old: males n = 2, females n = 2; *Rho*^*+/+*^: 4-month-old: males n = 2, females n = 2; Fig. 3B).

To enable easy and low-cost measurement of the OMR, we developed an open-source evaluation system of the mouse OMR (Fig. 1). The system was constructed with two components: (1) stimulus presentation and (2) mouse motion monitoring (Fig. 1A). For the stimulus presentation, four 21.5-inch full HD monitors (1920 ×1080 pixels, 60 Hz refresh rate, 250 cd/m² brightness; LG Monitor series; dimensions with stand: 492 × 378 × 200 mm) were placed surrounding a mouse at the center of the screens, which formed an evaluation chamber (56 × 56 cm wide, 37 cm height). The mouse was placed on a 3D-printed platform (base diameter = 150 mm, top diameter = 53 mm, height = 155 mm) to adjust a vertical viewing angle and restrict the mouse motion only to rotation. Stimulus presentation was controlled by a custom-made script (available at https://github.com/ssoma23/OMR_project.git) written in Python with PsychoPy module [18] which was integrated in a graphical user interface (GUI) with functionality of an online parameter update of grating stimulus (see below). Mirrors were placed at the bottom and top of the evaluation chamber to achieve the nearly full-field presentation of the grating stimulus. For the linearity of a gray-scale stimulus presentation, screens were gamma corrected with a luminance meter (LS-110, Konica Minolta; measured gamma = 2.029). This procedure was also conducted with a custom-made Python script (available at https://github.com/ssoma23/OMR_project.git).

**Fig. 1:**
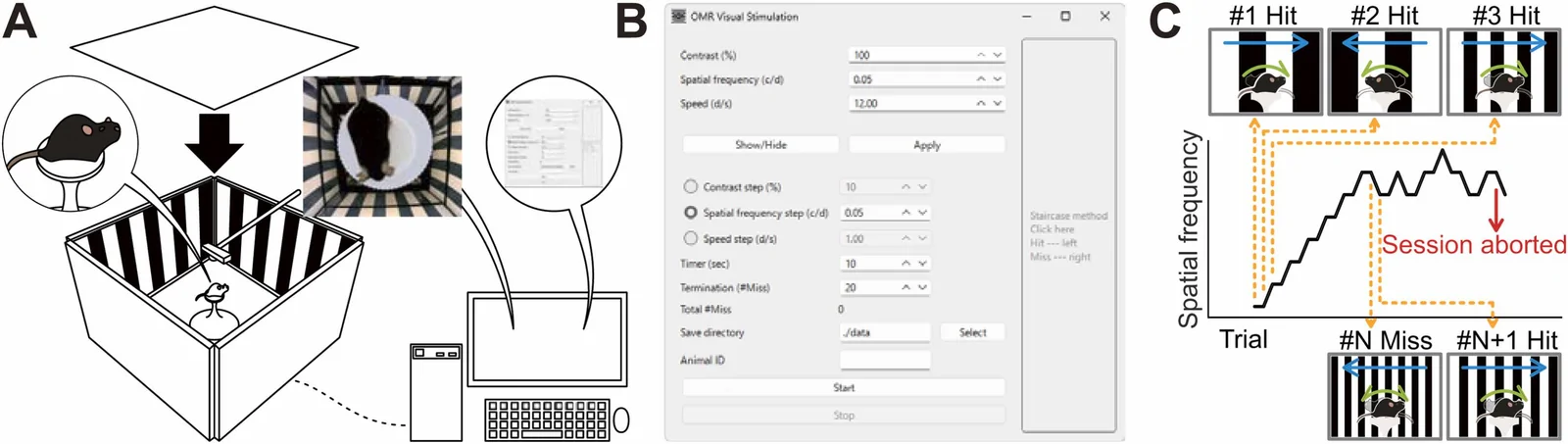
Scheme for optomotor response (OMR) measurement. **(A)** Overview of the whole OMR evaluation system which consists of four screens surrounding a mouse, mirrors providing a nearly full-field stimulus presentation, a platform the mouse is placed on, and a camera monitoring the mouse motion. Evaluators screen shows the mouse motion taken from the top and a control software **(B)** which provides the controls of multiple parameters of grating stimulus and update function of the grating parameter upon the mouse OMR. Our system offers three operating modes for measuring contrast, spatial frequency (SF), and speed. **(C)** A parameter of the grating stimulus (e.g., SF) was updated according to the mouse response in the manner of the staircase method. When the mouse follows the grating direction, the direction is flipped (after first hit) or stimulus parameter is updated to a one-step harder value (e.g., higher SF, after two consecutive hits). If no OMR was observed within the defined time window, the stimulus parameter was updated to a one-step easier value (e.g., lower SF). After five total misses, a session is aborted and the threshold for inducing the mouse OMR is determined based on the evaluated parameters.

For mouse motion monitoring, a webcam (1920 ×1080 pix, 60 FPS, W-04cam, Goseare) was placed at the top of the evaluation chamber, 17 cm above the 3D-printed platform. Real-time mouse motion was monitored and recorded with a standard camera application on Windows 11. An evaluator could watch the mouse motion and update the stimulus parameter by clicking a dedicated area on the GUI (Fig. 1B). The stimulus parameter was updated in the manner of the staircase method (Fig. 1C). When the mouse's head moved in the direction of the grating stimulus, the trial was classified as a "hit" (OMR presence; Supplementary Video 1), and the evaluator clicked the left mouse button. Depending on the trial history, this action either flipped the grating direction—specifically after the initial hit or a hit immediately following a miss—or automatically updated the grating parameter to a more challenging condition (e.g., higher spatial frequency [SF]). If the mouse’s head failed to move in the direction of the grating stimulus within a predetermined trial duration (10 s in this study), the trial was classified as a "miss" (OMR absence; Supplementary Video 1), and the grating parameter was automatically updated to a less challenging condition (e.g., lower SF). For SF measurements, the initial and minimum values were set to 0.15 and 0.05 cycles/degree, respectively. All mice were subjected to OMR evaluation without pre-test exclusion. Gross ocular inspection (e.g., to record age-related corneal opacity) was performed post hoc after the behavioral assays were completed. An animal was classified as "ND" (Not Detected) if it failed to exhibit a stimulus-locked OMR under baseline conditions during the test. In SF measurement mode (Fig. 3), "ND" was defined as the complete absence of OMR at the minimum SF of 0.05 cycles/degree under 100% contrast. In contrast sensitivity measurement mode (Fig. 2D), "ND" was defined when no OMR was observed at 100% contrast for a given SF. Parameters in each trial were recorded in a CSV file. When the total miss number reached a criterion, a session was aborted. The final stimulus level (maximum SF or minimum contrast) at which the mouse showed OMR in both clockwise and counter-clockwise trials was adopted. An evaluator could configure the initial stimulus parameters, step size for updating in the staircase method, trial duration, and total miss number for the criterion of session abortion via GUI (Fig. 1B).

**Fig. 2:**
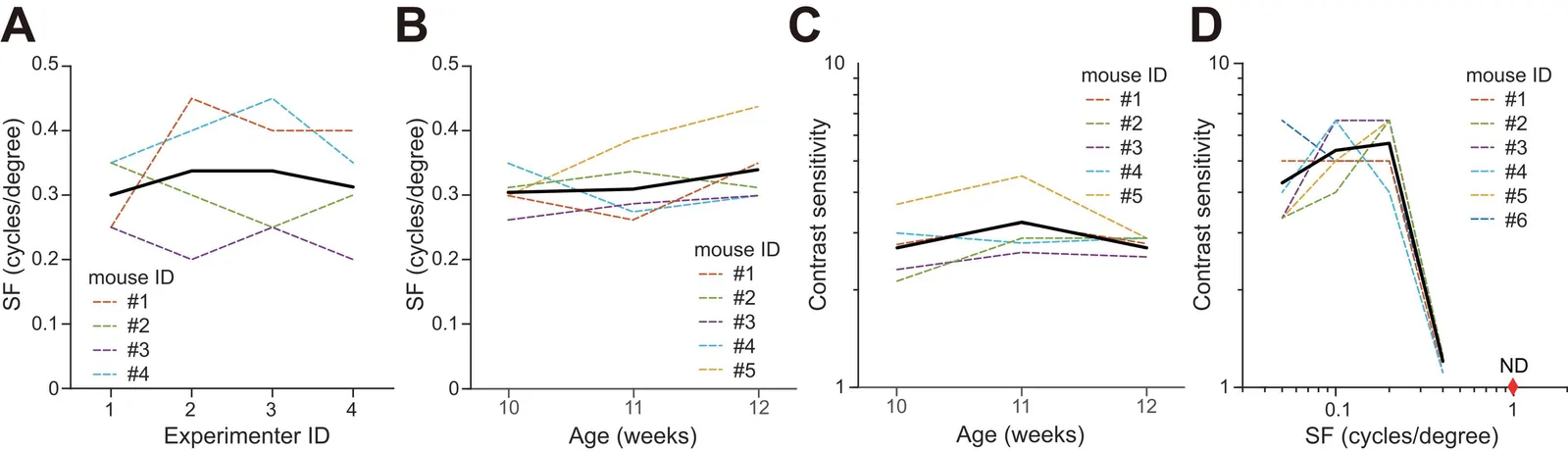
Reliability and validity of OMR-based visual function measurements. **(A)** Inter-observer reliability of SF measurements among four experimenters. There were no significant differences in SF values across the four experimenters. (P = 0.765, F_(3,9)_ = 0.388, one-way repeated measures ANOVA). **(B, C)** Week-to-week stability of SF and threshold contrast. All five tested mice show no significant difference in these parameters across three weeks (SF measured at 100% contrast: one-way repeated-measures ANOVA, F_(2,8)_ = 1.02, P = 0.40; contrast sensitivity measured at SF of 0.05 cycles/degree: one-way repeated-measures ANOVA, F_(2,8)_ = 1.91, P = 0.21). **(D)** Contrast sensitivity of wild-type mice. The OMR system yielded contrast sensitivity results comparable to those obtained with other established measurement systems. The black line indicates the mean. Red diamonds indicate not detectable (ND).

Experimenters were trained to reliably recognize the OMR in mice through a two-step protocol. First, trainees were instructed to identify typical OMR patterns using a demonstration video (Supplementary Video 1). Emphasis was placed on distinguishing the characteristic small, slow head and neck movements of the OMR from rapid, large whole-body movements. Next, experimenters practiced real-time OMR detection in live mice under the direct supervision of an expert. This supervised training protocol typically required less than one hour to complete.

## Results

To evaluate inter-evaluator reliability of our system, four evaluators independently measured visual function in four mice. We first trained each evaluator to detect the OMR of a mouse for around one hour. Then, we assessed inter-evaluator variability on the estimated visual acuity (maximum SF the mouse exhibited OMR) with our system (Fig. 2A, timeout = 10 s, abortion criteria = 5 Misses). We found no significant difference in the estimated visual acuity across the evaluators (P = 0.765, F_(3,9)_ = 0.388, one-way repeated measures ANOVA). In addition, we tested week-to-week stability of SF and contrast sensitivity. The same experimenter tested five mice across three weeks. SF and threshold contrast did not differ significantly across the three weeks (SF: one-way repeated-measures ANOVA, F_(2,8)_ = 1.02, P = 0.40; contrast sensitivity: one-way repeated-measures ANOVA, F_(2,8)_ = 1.91, P = 0.21). These results indicate that our system can evaluate the visual acuity of normal-sighted mice independent of evaluators as well as the measurement period. Another commonly used measure of visual ability is contrast sensitivity, defined as the inverse of the minimum detectable contrast. By changing the measurement mode, our system can also evaluate contrast sensitivity. To validate whether our system accurately captures the low-pass contrast sensitivity function, which is reported previously [9], [11], [13], [19], [20], [21], [22], [23], we measured contrast sensitivity across multiple SFs. The resulting contrast sensitivity function (Fig. 2D, n = 6) showed comparable results with the previous studies.

In addition to evaluating visual acuity in healthy animals, our system could be utilized to assess age-related declines in visual acuity of wild-type mice (Fig. 3A). Specifically, 18-month-old mice exhibited significantly low barely-detectable SF compared with 9-month-old mice (P = 2.5005e-06, t_(17)_ = 6.915, unpaired *t*-test; Note that older mice failing to exhibit an OMR were excluded from this analysis [n = 2; ND]).

**Fig. 3:**
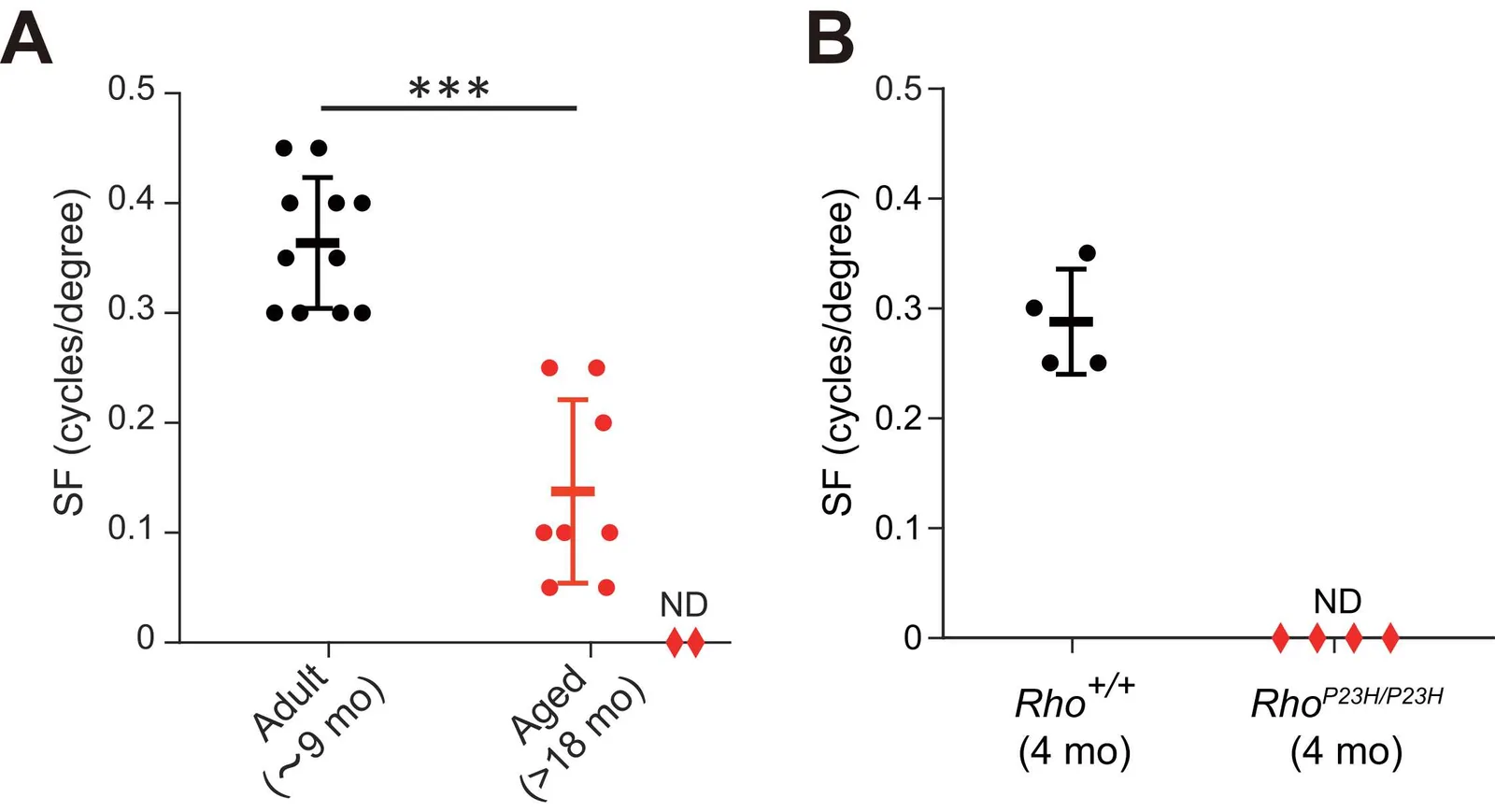
Visual acuity measurements in aged mice and retinitis pigmentosa model mice. **(A)** Comparison of visual acuity between adult (~9-month-old) and aged (>18-month-old) wild type mice (P = 2.5005e-06, t_(17)_ = 6.915, unpaired *t*-test). Each dot represents an individual mouse (Adult: n = 11, Aged: n = 8). Mice marked with a red diamond represent aged mice with corneal opacity that failed to exhibit observable OMR at any SF (ND, n = 2). **(B)** While SF was successfully measured in *Rho*^*+/+*^ mice (4-month-old), it was undetectable in littermate *Rho*^*P23H/P23H*^ mice due to the advanced stage of retinal degeneration. Each dot represents an individual mouse (*Rho*^*+/+*^: n = 4, *Rho*^*P23H/P23H*^: n = 4). Red diamonds indicate not detectable (ND).

As another validation of our system, we evaluated the visual acuity of the retinitis pigmentosa model mice (*Rho*^*P23H/P23H*^), which exhibited no visible signs of OMR to any grating stimulus while the age-matched littermates showed robust OMRs (Fig. 3B).

Thus, our system is applicable to evaluating visual function in young and adult wild-type mice as well as in mouse models of age-related or genetically induced visual decline.

## Discussion

Our open-source OMR-recording system offers a low-cost, expandable platform for evaluating visual acuity and contrast sensitivity, requiring minimal evaluator training. Our system can be constructed for less than $3000, which is substantially less expensive than commercial alternatives. This reduces the financial barriers and enables researchers to readily adopt OMR-based visual assessments.

Beyond cost efficiency, our platform introduces key methodological and design advantages over existing setups. First, the streamlined GUI provides an intuitive interface for real-time parameter configuration—such as measurement mode, stimulus duration, SFs, and contrast levels—making it accessible to non-programmers. Second, the control software is written in Python/PsychoPy, offering open-source flexibility for advanced protocol customization. Third, the compact physical footprint (56 × 56 cm chamber) allows easy placement on standard laboratory benches without requiring dedicated rooms, which is an important factor for scaling up high-throughput experiments. Fourth, combining an untrained reflex assay with a standardized, brief evaluator training protocol minimizes experimental setup time and ensures high reproducibility across different operators (Fig. 2A) and testing sessions (Fig. 2B,C).

Visual function assessment based on OMR is widely utilized for evaluating the intervention effects such as AAV-mediated gene therapy [24], [25], [26]. In addition to therapeutic studies, the OMR assessment is also used in the field of vision study using normal-sighted mice, including visual acuity assessment and contrast sensitivity assessment [14], [15], [16], [17]. Our system can also measure temporal frequency tuning by changing the measurement mode, which is a parameter commonly assessed in both basic and disease-related studies [12], [23], [24]

Our open-source OMR-recording system overcomes the disadvantage related to the introduction cost, and can be applicable to a wide range of studies like the assessment of optokinetic response [15], [27], visual evoked potential [3], and neural spiking activity [11], [28] as well as studies using other species like fishes [29]. We hope that our system can expand and accelerate the therapeutic and physiological studies demanding assessments of visual functions.

A limitation of the present system, shared with other previously reported visual setups [13], [14], [16], [17], [22], is its reliance on the subjective judgment of the evaluator, which may potentially introduce inter-evaluator variability. To mitigate this potential variability, we established explicit behavioral definitions for 'Hit' and 'Miss' events, supported by an instructional video (Supplementary Video 1). Within our study, this brief one-hour training workflow effectively aligned decision thresholds across operators, yielding high inter-evaluator reproducibility without necessitating extensive prior experience (Fig. 2A). Nevertheless, as direct comparative benchmarks with conventional systems were not conducted in this study, further validation of this standardized training protocol across different laboratories will be valuable to support its broader adoption.

In the present version, we intentionally prioritized manual evaluation by human experimenters to keep system setup and operational complexity to a minimum. Although real-time pose tracking tools (e.g., Kane et al., 2020 [30]) can run on standard CPUs using lightweight neural networks, automating OMR detection requires more than tracking keypoint coordinates; it necessitates a validated behavioral classifier to accurately discriminate stimulus-locked tracking from spontaneous head movements (e.g., grooming or voluntary exploration). While automated tracking systems have been established (e.g., Kretschmer et al., 2015 [15]), our human-in-the-loop approach—supported by a standardized 1-hour training protocol—achieves high inter-evaluator reproducibility without requiring custom classifier training or specialized hardware setups. Nevertheless, because our system is built entirely on an open-source Python framework, integrating pose estimation packages combined with automated behavioral classification algorithms represents an attractive modular upgrade for future fully automated applications.

## CRediT authorship contribution statement

**Naofumi Suematsu:** Writing – review & editing, Writing – original draft, Software, Methodology, Investigation, Formal analysis, Conceptualization. **Toshihide Ikeda:** Writing – review & editing, Methodology, Investigation, Formal analysis. **Takanori Aoki:** Writing – review & editing, Methodology, Investigation, Funding acquisition, Formal analysis. **Moe Asayama:** Writing – review & editing, Methodology, Investigation, Formal analysis. **Takuro Horii:** Writing – review & editing, Resources, Funding acquisition. **Atsushi Hoshino:** Writing – review & editing, Supervision, Project administration, Funding acquisition. **Hiroshi Tanaka:** Writing – review & editing, Supervision, Project administration. **Shogo Soma:** Writing – review & editing, Writing – original draft, Supervision, Project administration, Investigation, Funding acquisition, Formal analysis, Conceptualization.

## Declaration of Generative AI and AI-assisted technologies in the writing process

The authors used Claude for proofreading the manuscript, and thus take full responsibility for the content of the publication.

## Funding

This work was supported by Grant-in-Aid for Research Activity Start-up (JP25K24022 to T.A.), Grants-in-Aid for Scientific Research (B) (JP26K02836 to S.S.), Challenging Research (Exploratory) (JP26K23618 to S.S.) and Transformative Research Areas (A) (JP25H01746, JP25H02624 to S.S.) from 10.13039/501100001691JSPS; by JST FOREST Program, (JPMJFR251Z to S.S.); by the Japan Agency for Medical Research and Development (AMED) (JP24ek0109711h0001 to A.H.); the Platform Project for Supporting Drug Discovery and Life Science Research (Basis for Supporting Innovative Drug Discovery and Life Science Research (BINDS)) from AMED (JP24ama121049 to T.H.); and by the 10.13039/100007449Takeda Science Foundation (S.S.); by the Lotte Research Promotion Grant (S.S.); by the Research Foundation for OptoScience and Technology (S.S.).

## Declaration of Competing Interest

The authors declare the following financial interests/personal relationships which may be considered as potential competing interests: Shogo Soma reports financial support was provided by Japan Society for the Promotion of Science. Shogo Soma reports financial support was provided by Japan Science and Technology Agency. Atsushi Hoshino reports financial support was provided by Japan Agency for Medical Research and Development. Takanori Aoki reports financial support was provided by Japan Society for the Promotion of Science. Shogo Soma reports financial support was provided by Takeda Science Foundation. Shogo Soma reports financial support was provided by Research Foundation for OptoScience and Technology. If there are other authors, they declare that they have no known competing financial interests or personal relationships that could have appeared to influence the work reported in this paper.

## Data Availability

The datasets presented in this article are available at https://github.com/ssoma23/OMR_project.git.

## Appendix A Supplementary material

**Supplementary Video 1. Representative session of spatial frequency measurement using the OMR system.** This video demonstrates a representative single session of spatial frequency (SF) threshold measurement. The session begins at an initial SF of 0.05 cycles/degree (cpd), with the mouse exhibiting optomotor responses (OMR) up to a maximum SF of 0.4 cpd. The main full-screen video is presented at normal speed (1 ×), displaying a "Hit" annotation whenever a stimulus-locked OMR is elicited. Representative OMR events are also replayed in the inset window, with the corresponding playback speeds indicated on-screen. In contrast, trials classified as a "Miss" show no observable OMR within the designated time window (10 s), after which the stimulus parameter is automatically updated to a less challenging condition (e.g., lower SF)
